# Re-Sensitizing Cancer Stem Cells to Conventional Chemotherapy Agents

**DOI:** 10.3390/ijms24032122

**Published:** 2023-01-20

**Authors:** Mariyam Kim, Laura Bakyt, Azamat Akhmetkaliyev, Dana Toktarkhanova, Denis Bulanin

**Affiliations:** Department of Biomedical Sciences, Nazarbayev University School of Medicine, Astana 010000, Kazakhstan

**Keywords:** cancer stem cells, signaling pathways, chemoresistance, cancer therapeutics

## Abstract

Cancer stem cells are found in many cancer types. They comprise a distinct subpopulation of cells within the tumor that exhibit properties of stem cells. They express a number of cell surface markers, such as CD133, CD44, ALDH, and EpCAM, as well as embryonic transcription factors Oct4, Nanog, and SOX2. CSCs are more resistant to conventional chemotherapy and can potentially drive tumor relapse. Therefore, it is essential to understand the molecular mechanisms that drive chemoresistance and to target them with specific therapy effectively. Highly conserved developmental signaling pathways such as Wnt, Hedgehog, and Notch are commonly reported to play a role in CSCs chemoresistance development. Studies show that particular pathway inhibitors combined with conventional therapy may re-establish sensitivity to the conventional therapy. Another significant contributor of chemoresistance is a specific tumor microenvironment. Surrounding stroma in the form of cancer-associated fibroblasts, macrophages, endothelial cells, and extracellular matrix components produce cytokines and other factors, thus creating a favorable environment and decreasing the cytotoxic effects of chemotherapy. Anti-stromal agents may potentially help to overcome these effects. Epigenetic changes and autophagy were also among the commonly reported mechanisms of chemoresistance. This review provides an overview of signaling pathway components involved in the development of chemoresistance of CSCs and gathers evidence from experimental studies in which CSCs can be re-sensitized to conventional chemotherapy agents across different cancer types.

## 1. Introduction

Despite significant progress in cancer therapy with conventional anti-cancer chemotherapy agents, a substantial portion of patients experiences disease progression, metastasis, and relapse [1]. For decades, tumor formation and propagation mechanisms were explained by the stochastic clonal expansion model, proposed in 1976 by Dr. Nowell, according to which cells accumulate mutations with time and the one with the most aggressive phenotype drives tumor formation [2]. Hence, any of the tumor cells may potentially transform into malignant ones. Evidence suggests that this model may be accurate for several cancers. However, it is still far from being ideal. A growing body of evidence suggests a more plausible hierarchical model in which different populations of cells exist within a tumor with variable potency of tumorigenesis [3]. The most potent fraction exhibits stem cell-like properties of self-renewal and differentiation capacity called cancer stem cells (CSCs) [4]. These cells are theoretically able to regenerate the parental tumor. The hierarchical theory was first proposed after identifying leukemia-initiating cells (LICs) in acute myeloid leukemia. LICs represented only a small fraction of cells (0.2–100/106 leukemic blasts) but were able to re-establish tumors in immunodeficient mice [4]. This work started the interest in exploring the presence of CSCs in other hematological and solid malignancies. In 2001, brain CSCs were identified, followed by breast, melanoma, and many other cancers [2]. To date, CSCs are still being determined.

Distinct cell surface markers are used as a surrogate to identify cells with functional properties of CSCs. CD133 or prominin-1, a transmembrane glycoprotein, is used to select highly chemo- and radioresistant glioma cells subset [3]. Expression of CD133 was also a negative predictor of the outcome for patients. Interestingly, overexpression of CD133 turned hepatocellular carcinoma cells, prostate cancer cells, and melanoma cells into CSCs.

Breast CSCs highly express CD44 and are commonly highly resistant to radiotherapy, likely due to enhanced free radical scavenging mechanisms [5]. The same marker was proposed for head and neck CSCs identification; however, its use is more controversial. Recent evidence suggests that a combination of CD44 and CD133 in colorectal cancer is associated with a sevenfold increase in tumorigenicity compared to CD133 alone (1.45 times). Gastric cancer cells expressing CD44 showed enhanced self-renewal capacity and, interestingly, could give rise to a subpopulation of CD44-cells [5]. Aldehyde dehydrogenase appears to be more uniformly expressed by different CSCs. The Mammalian ALDHs family consists of 18 isotypes. It was initially proposed to be highly expressed in hematopoietic stem cells but later was identified in breast CSCs, head and neck squamous cell carcinoma, gliomas, etc. [5,6,7]. ALDH overexpression was shown to be linked to poor outcomes [8].

EpCAM is another surface marker and a glycoprotein expressed on healthy epithelial cells. It gained recognition for identifying CSCs of breast, colon, hepatocellular and pancreatic cancers [8]. As CSCs closely resemble normal stem cells, it is not surprising that embryonic transcription factors such as Oct4, Nanog, and SOX2 may be re-activated and considered internal markers of CSCs [9]. Oct4+ cells exhibit more stem cell-like properties, such as enhanced self-renewal, tumorigenicity, and chemotherapy resistance. It was used to identify CSCs in breast, non-small cell lung cancer, gastrointestinal cancers, and hepatocellular cancer [10]. Nanog is generally required to support pluripotency and is downregulated in differentiated cells. Evidence suggests that Nanog expression correlates directly with the activity of cancer stem cells in non-small cell lung cancer, functioning like a switch between CSCs and differentiated cancer cells [11]. Sox2 is essential for early embryonic development and supports stem cells’ undifferentiated state. In cancer stem cell research, it is considered a marker of stemness. Sox2 was proposed to be used to identify bladder cancer stem cells as well as non-small cell lung carcinomas [12].

Numerous studies claim that CSCs play a critical role in cancer propagation, recurrence, and chemotherapy resistance [6,7]. Cancer stem cells are inherently more resistant to chemotherapy than their differentiated counterparts. Traditional chemotherapy targets the bulk of differentiated cancer cells, effectively reducing the tumor mass but selecting highly resistant CSCs that can regenerate the tumor. These cells then drive tumor relapse and are essential constraints to a disease-free state. Studies show that radiation therapy triggers the upregulation of embryonic transcription factors such as Sox2, Nanog, and Oct4 in breast cancer cells and induces a stem cell-like state [6,7]. Consistent with this observation, another study reported similar findings in hepatocellular carcinoma cells [13]. Understanding mechanisms driving chemoresistance is crucial for effectively targeting CSCs and sensitization to existing chemotherapy. This review will provide an overview of commonly reported mechanisms possessed by cancer stem cells that are implicated in chemotherapy resistance and discuss current target-specific therapies.

In addition, this article aims to provide an information about mechanisms and specific components of the signaling pathways to be involved in the development of chemoresistance of CSC. Moreover, it summarizes existing experimental studies demonstrating how pathway inhibitors may be used to re-sensitize CSCs to conventional chemotherapy by targeting particular pathway and cell environment components.

## 2. Notch Signaling Pathway

As an evolutionarily conserved route, the Notch signaling pathway plays a crucial role in the regulation of communication between neighboring cells during different stages of embryogenesis, differentiation, and apoptosis [14]. Notch signaling operates through the interaction of four Notch receptors (1–4) with two distinct families of ligands, Jagged (1–2) and Delta-like ligands (1, 3–4). Notch receptor, a heterodimer, contains extracellular, transmembrane, and intracellular domains. Upon binding the ligand, the receptor undergoes a conformational change with subsequent activation of the two-step proteolytic cleavage. Disintegrin, metalloproteinase (ADAM), and γ-secretase mediate the first and second cleavage, respectively. The latter releases an active intracellular domain, which translocates to the nucleus and regulates target gene expression [15]. Well-studied targets of the Notch pathway, Hes-1 and Hey-1, play an essential role in cell fate decisions to commit to certain cell types. Its functions were shown to be implicated in different aspects of cancer biology: promotion of angiogenesis, metastasis, evasion of the immune system, and promoting and maintaining the stemness of CSCs in certain cancers including but not limited to esophageal, skin squamous cell carcinomas, colorectal, lung, certain B- and T-cell leukemias, and breast cancer [16].

Vinson et al., reported that compared to the general pool of cancer cells, CSCs of colorectal cancer showed an increase of Notch signaling for up to 10–30 times with upregulation of HES1, Notch-1, and Jagged-1 expression [17]. Therefore, it was proposed to promote self-renewal through interaction with p27 and migration through involvement in epithelial-to-mesenchymal transition (EMT) via negative regulation of E-cadherin and β-catenin expression [18]. Moreover, Notch directly regulates c-Myc and cyclin D expression, which are known to be cell-cycle regulators [19]. Consistent with these findings, studies targeting HES-2 with shRNA resulted in a loss of stem cell-like properties and further differentiation of CSCs. This may indicate that Notch negatively regulates differentiation in breast cancer cells, resulting in the maintenance of stem cell state contributing to tumor formation [19].

Recent evidence suggests that the Notch pathway contributes to chemoresistance as well. Exposure of breast cancer cell lines to doxorubicin, docetaxel, and therapy with selective estrogen receptor modulators (SERMs) resulted in selecting a chemoresistant population of cells with increased expression of Notch 1 and Notch 4 [20]. Similarly, targeting Notch 1 with Psoralidin, these cells exhibited suppressed growth and increased apoptosis in animal models [20]. These findings are consistent with the work of Below and colleagues, who suggest that ovarian CSCs resistance is driven by Notch [21]. Inhibition of this pathway resulted in the sensitization of cancer cells to cisplatin and suppressed stemness. It was also demonstrated that the upregulation of Hes1 is responsible for stem cell-like properties of ovarian CSCs and subsequently enhanced chemotherapy resistance [22]. Additionally, targeted inhibition of the Notch pathway restored the sensitivity of lung CSCs to Gefitinib [23]. All of these findings suggest that inhibition of the Notch pathway may help to sensitize CSCs and increase the efficacy of classical anti-cancer therapy. Additional details of the Notch signaling pathway components in chemoresistance of CSCs is shown below in Table 1.

**Table 1:** Summary of evidence for the role of Notch signaling in chemoresistance of cancer stem cells.

Cancer Type	Inhibitor	Experimental Evidence	References
Colorectal cancer	DAPT, a gamma-secretase inhibitor	The decreased growth of 5-FU and oxaliplatin-resistant cells in vivo and in vitro	[24]
	DLD-1 and DAPT, compound E	Sensitized and significantly enhanced taxane-induced mitotic arrest and apoptosis both in vitro and in vivo	[25]
	GSI-34, a gamma-secretase inhibitor	Significantly sensitized the cells to oxaliplatin- and 5-fluorouracil through apoptosis	[26]
	DLL4 inhibitor	Enhanced oxaliplatin action and decreased activity of prosurvival pathways. Combination with irinotecan treatment reduced the frequency of CSCs	[27]
Medulloblastoma	Gamma-secretase inhibitor-18	Induced apoptosis in vitro in nestin-positive medulloblastoma cells with stem cell-like properties	[28]
Ovarian cancer	GSI, siRNA	Dramatically increased platinum-based therapy sensitivity both in vivo and in vitro	[29]
	shRNA knockdown	Resulted in the sensitization of ovarian cancer cells exhibiting stem cell markers to carboplatin in vitro	[30]
Insulinoma	DAPT	Reversed resistance to 5-FU in vitro tumor proliferation in vivo was significantly decreased when the drugs were used in combination compared to their use as single agents	[31]
Breast cancer	GSI	Enhanced doxorubicin antitumor activity in vitro and in vivo	[32]
	Psoralidin	This resulted in growth inhibition and induction of apoptosis in breast CSCs resistant to doxorubicin	[22]
	Notch1 monoclonal antibodies	Sensitized triple-negative breast CSCs to docetaxel	[33]
Esophageal cancer	siRNA	Reduced levels of 5-FU resistance in vivo	[34]
Prostate cancer	GSI	Enhanced the antitumor effect of docetaxel in prostate cancer stem-like cells	[35]
	siRNA, compound E, GSI	Depleted chemoresistant prostate cancer-initiating cells in vitro and in vivo	[36]

## 3. The Wnt Pathway

The Wnt signaling pathway is another important signaling pathway that plays a role in embryonic development, maintenance of tissue homeostasis, self-renewal, as well as differentiation [37]. There are three main WNT pathways: the canonical, involving β-catenin, T cell-specific transcription factor (TCF), and lymphoid enhancer-binding factor (LEF), non-canonical—β-catenin independent, and non-canonical responsible for intracellular calcium regulation [38]. In humans, the Wnt family comprises 19 secretory lipid-modified glycoproteins, which interact with around ten isoforms of Frizzled receptors and different co-receptors, mainly low-density receptor-related protein 5/6 [38]. Interaction with ligands in the canonical pathway determines the stability of the critical player β-catenin [39]. In the absence of a ligand, β-catenin is phosphorylated and further undergoes degradation by the proteasome. However, upon ligand binding, β-catenin escapes destruction and accumulates in the cytoplasm. This allows subsequent β-catenin translocation to the nucleus and activation of transcription complex consisting of T-cell specific transcription factor and enhancer-binding factor (TCF-LEF). As a result, genes involved in cell fate determination and proliferation are differentially regulated. Given the function, it is not surprising that the WNT pathway is one of the most mutated pathways of all, and it contributes to the formation of solid and hematological malignancies, including colorectal, hepatocellular cancers, melanoma, AML, CLL, etc. [40,41]. Several constituents of the Wnt pathway, including LEF1, TCF-4, β-catenin, and cyclin D1, are upregulated in the CSCs compared to non-CSCs [42]. Both canonical and non-canonical WNT pathways were reported to play important roles in developing and maintaining CSCs and chemotherapy resistance. Pancreatic CSCs resistant to 5-FU showed aberrant regulation of the WNT pathway [43]. Pery and colleagues stated that leukemia stem cells with upregulated WNT pathway expanded under the stress of conventional chemotherapy, whereas targeted inhibition resulted in reduced tumorigenic activity [44]. Similar findings were reported in breast cancer studies. Inhibition of WNT signaling reduced the burden of self-renewal properties of CSCs, decreased expression of CSCs markers ALDH and CD44, and suppressed growth in vitro and in vivo [44]. Interesting findings were reported by Martins-Neves et al., who showed that low-dose doxorubicin used for the treatment of osteosarcoma, induced increased WNT signaling, measured by TCF/LEF-luciferase transcriptional activity and exhibited resistance. When treated with a WNT pathway inhibitor, these cells had a significantly decreased cell viability and decreased expression of stem cell markers [45]. Chemoresistant CD 133+ neuroblastoma cells were also reported to upregulate the Wnt pathway upon treatment with doxorubicin, which was proposed to contribute to enhanced survival. In the presence of pathway inhibitors, the number of live cells in the chemoresistant group significantly dropped [46]. Further, the role of different inhibitors in chemoresistance of CSCs are shown in Table 2.

**Table 2:** Summary of evidence for the role of Wnt signaling in chemoresistance of cancer stem cells.

Cancer Type	Inhibitor	Experimental Evidence	References
Ovarian cancer	XAV-939	Effectively reversed cisplatin chemoresistance in vitro	[47,48]
	CCT036477	Inhibition of β-catenin transcriptional activity sensitized previously resistant cells to carboplatin in vitro	[49]
	ICG-001	Sensitized cells to cisplatin and decreased the number of cancer-initiating cells	[50]
Neuroblastoma	XAV-939 and ICG-001	Combination treatment with doxorubicin enhanced cytotoxicity against cancer stem-like cells	[49]
	XAV-939	Significantly enhanced the sensitivity of cells to doxorubicin in both 2D and 3D culture systems	[51]
Glioma	sFRP4	Increased apoptosis in combination with doxorubicin/cisplatin and significantly decreased number of glioma stem cells	[52]
Colon cancer	XAV939	Significantly increased apoptosis induced by 5-FU/DDP and decreased expression of stemness markers in vitro	[53]
	RNAi of TCF4	Significantly sensitized CRC cells to radiotherapy	[54]
	IC-2	Reduced sphere numbers of CD44 high cells and sensitized CSCs to 5FU in vitro	[55]
Head and neck squamous cell carcinoma cells	XAV939	Combination with cisplatin acted synergistically to abrogate chemoresistance by increasing DNA damage in cells with CSCs phenotype	[56,57]
	HC-1	Sensitized and significantly enhanced the cytotoxicity of 5-FU in CD44 CSCs	[58]
	sFRP4	Inhibited HNSCC proliferation and increased efficacy of doxorubicin and cisplatin via an increase in apoptosis	[59]
Hepatocellular carcinoma	Lentiviral miRNA against β-catenin	Significantly diminished growth of cisplatin chemoresistant colonies consisting of progenitor-like cells	[60]
Nasopharyngeal carcinoma	ICG-001	Effectively inhibited the growth of a CSC-like population in combination with cisplatin	[61]
ALL	ICG-001	Induced differentiation of ALL CSCs and sensitized them to VDL chemotherapy	[62]
CML	ICG-001	Eliminated drug-resistant CML leukemia-initiating cells and sensitized resistant cells to imatinib	[63]
Breast cancer	ICG-001	Decreased emergence of drug-resistant, highly aggressive cancer stem-like phenotype in vitro	[64]
	CWP232228	Affected chemoresistant BCSC maintenance	[65]

## 4. Hedgehog Signaling Pathway

The Hedgehog (Hh) signaling pathway initially identified in *Drosophila melanogaster* regulates essential processes during embryonic development, such as proliferation, survival, patterning, migration, and cell fate determination by altering protein trafficking, gene expression, and protein–protein interactions [66]. However, the HH pathway is subsequently silenced later in development in most tissues except for limited organ systems such as the central nervous system, the lungs, theca, and Leydig cells, which continue to rely on it for repair and homeostasis [67].

There are three primary HH homologs, namely Sonic Hedgehog (Shh), Indian Hedgehog (Ihh), and Desert Hedgehog (Dhh), that differ in the pattern of spatial and temporal expression and distribution. They subsequently undergo maturation through several autoproteolytic steps and covalent modifications before an active ligand is released [68]. Signaling is initiated when a ligand binds to a 12-pass transmembrane receptor called Patched (PTCH1), which in the absence of a ligand, represses GPCR-like protein Smoothened (SMO). Upon binding of HH ligand, SMO translocate to the plasma membrane and subsequently weakens the interaction of suppressor of fused homolog (Sufu) and glioma-associated oncogene homolog (Gli) transcription factors [69]. This alters the expression of Gli family proteins’ target genes such as SNAIL, c-MYC, BCL-2, and Prominin-1 (CD133) [70].

Given its many roles and target genes, it is not surprising that dysregulation of the pathway in a lifetime can potentially contribute to cancer development. HH pathway was proposed to be responsible for a cell population with stem cell-like properties, supporting self-renewal capacities and upregulating stemness-determining genes [69,70]. One of such master determinants, Nanog, is directly targeted by the HH pathway. Moreover, it drives expressions of Oct4, Bmi1, and SOX2 [71]. Numerous studies reported that small molecule pathway inhibitor cyclopamine effectively reduced self-renewal potential, promoted apoptosis, and prevented relapse in different cancers, including acute and chronic myeloid leukemia, breast cancer, small and non-small cell carcinoma, and pancreatic and prostate cancer [72,73,74,75].

It was also proposed to be implicated in cancer stem cell resistance to chemotherapy in different cancer types [76,77,78,79,80,81,82,83,84,85,86,87,88,89]. Inhibition of HH pathway was achieved in cyclopamine-sensitized paclitaxel-resistant breast cancer cells in vitro, presumably through antagonizing Smo action. Moreover, the tumor size decreased in the xenograft model [76]. A similar observation was reported in doxorubicin-resistant breast cancer cells, further supporting the idea of the HH pathway involvement in resistance development to conventional chemotherapy agents [77]. Another pathway inhibitor, GANT61, a derivative of hexahydro pyrimidone, selectively inhibits the action of GLI transcription factors. It was also demonstrated that GANT61 decreased resistance to the FOLFOX regimen in colorectal cancer cells [87]. Additionally, vismodegib, another selective inhibitor of the pathway, sensitized tamoxifen-resistant breast cancer cells and inhibited the growth of tumors in xenograft models [80]. Evidence supporting HH pathway implication in the chemoresistance of cancer stem cells is summarized in Table 3.

**Table 3:** Summary of evidence for the role of Hedgehog signaling in chemoresistance of cancer stem cells.

Cancer Type	Inhibitor	Experimental Evidence	References
Breast cancer	Cyclopamine	Enhanced paclitaxel-induced cell death in vitro and decreases tumor growth in a xenograft model	[76,77]
		Sensitized doxorubicin-resistant breast cancer cells evident from diminished tumor size in the xenograft model of nude mice	[78,79]
	Vismodegib	Inhibited growth of tumors in tamoxifen-resistant xenografts Downregulated CSC markers and sensitized cells to docetaxel	[80,81]
Pancreatic cancer	Cyclopamine	Restored gemcitabine sensitivity in gemcitabine-resistant cells	[82,83,84,85,86]
	Smo knockdown	Downregulated CSC markers	
	Gli1 shRNA	Downregulated CSC markers	
Colorectal cancer	GANT61	Decreased the resistance to 5-FU, irinotecan, and oxaliplatin	[87,88]
Glioblastoma multiforme	Cyclopamine	Cyclopamine potentiated temozolomide treatment in glioblastoma cell lines by inducing apoptosis	[89,90,91,92,93,94]
Prostate cancer	Cyclopamine	Enhanced paclitaxel-mediated growth suppression in previously resistant cells	[95,96]
Gastric cancer	Cyclopamine	Significantly improved the tumor response to the drug in oxaliplatin-resistant gastric CSCs	[97]
	Gli1 knockdown	Enhanced the efficacy of chemotherapy and significantly reduced self-renewing capacity	[98]

## 5. Microenvironment

Cancer stem cells share a lot of similarities with normal adult stem cells. However, the latter reside at locations less exposed to external stimuli and damage. For example, intestinal stem cells reside at the bottom of the crypt, mammary stem cells are away from the lumen of the acinus, and hematopoietic stem cells are shielded with the bone. Like the normal counterparts, CSCs have their protected microenvironments, sometimes called “niches” [99,100]. The niche is a three-dimensional structure constituted by collagens, various glycoproteins, and other ECM components which provide tissue architecture, allow communication and create a barrier [101]. Numerous studies proposed that niches are essential for regulating stem cell state, prevention of differentiation, and response to treatment via cell-to-cell or extracellular matrix components-to-cell communication [40,102]. The complex interplay between CSCs and ECM components, stroma, tumor-associated fibroblasts, and hypoxic state balances proliferation-promoting and inhibiting signals to maintain homeostasis. However, under environmental stress in the form of cytotoxic therapy, tumor cells, and the microenvironment may respond in various ways, which might lead to chemoresistance development.

A growing body of evidence supports the idea that hypoxia is a powerful driver of therapeutic resistance [103,104,105]. This was proposed to result from the upregulation of stemness developmental pathways discussed above. Alternatively, chemoresistance may be induced by miRNA regulation of hypoxia-inducible factors [103]. For example, according to Xiao et al., under hypoxic conditions, miR-520-f-3p promotes stem cell properties and resistance to sorafenib in hepatocellular CSCs [106]. Roscigno et al., reported similar results with colleagues, who showed that another miRNA, miR-24, regulates stemness and reduces chemotherapy-induced apoptosis in breast CSCs [107].

CSCs were also reported to form unique immunosuppressive niches under the pressure of chemotherapy through increased Granulocyte-Macrophage Colony-Stimulating Factor (GM-CSF) secretion from anti-inflammatory M2- macrophages [108]. At the same time, CSCs show enhanced production of pro-inflammatory cytokines such as IL-1β, IL-6, IL-8, and CCL2. The latter stimulates CCR2+ monocytes, which function as a precursor to the immunosuppressive microenvironment [109]. Similar to macrophages, fibroblasts may be converted to cancer-associated fibroblasts (CAFs) under the influence of TGF-β and platelet-derived growth factor (PDGF). Studies suggest that CAFs actively participate in resistance development via enhanced secretion of ECM components and metastasis through the production of fibronectin, periostin, and metalloproteinases, thereby acquiring mesenchymal properties [110].

Moreover, recent studies have shown that surrounding stroma consisting of endothelial cells and smooth muscle cells that nourish the tumor is commonly driven in a protumorigenic wound-healing state. Paracrine signaling and cross-talk in the niche promote the secretion of angiogenic factors and hence survival [111]. Ferguson et al., demonstrated that leukemia CSCs need CD98 for successful perivascular adhesion, and treatment with anti-CD98 antibody enhances response to therapy [112]. Similar results were obtained from another study, where aggressive chemoresistant leukemias were sensitized to tyrosine kinase inhibitors upon treatment with adhesion signal inhibitors [113]. Additional data were gathered on tumor microenvironment role in chemoresistance of CSCs in Table 4.

**Table 4:** Summary of evidence for the role of tumor microenvironment in chemoresistance of cancer stem cells.

Cancer Type	Intervention	Experimental Evidence	References
Glioblastoma multiforme	Hypoxia	Hypoxia induced increased expression of stemness markers (CD133, Sox-2, Bmi-1, podoplanin, nestin) and chemoresistance-associated markers (MGMT, MRP1, MDR-1, TIMP-1, Lamp1)	[114]
	Anti-GPR77 antibody	Reduced tumor formation and restored sensitivity to chemotherapy by targeting CD10+GPR77+ cancer-associated fibroblasts	[115]
Breast cancer	CAFs treated with Smo-i (HH inhibitor)	Reduced metastatic growth and sensitized to chemotherapy with taxanes	[86]
	HIF-2α overexpression	Induced expression of stem cell markers c-Myc, OCT4, and Nanog and the resistance to paclitaxel	[86]
	IL-6 antibody	Re-sensitized CSCs with acquired trastuzumab resistance	[116]
	IL4DM, IL-4 receptor antagonist	Combined with fulvestrant for ER+ CSCs and with docetaxel for triple-negative CSCs, potentiated cell death, and chemotherapeutic action	[117]
Colorectal cancer	Antibody against IL-17A	Augmented the cytotoxic efficacy of chemotherapy with 5-FU and Oxaliplatin	[118]
	Co-culture with CAFs secreting TGF-b2 and IL-6	Increased resistance to 5-fluorouracil/oxaliplatin due to upregulation of Gli2	[93]
	MFG-E8 produced by tumor-associated macrophages	The presence of MFG-E8 suppressed cisplatin-induced caspase-3 mediated apoptosis in vitro	[119]
Melanoma	Hypoxia	Promoted partial resistance to dacarbazine, increased self-renewal capacity, and promoted invasion through upregulation of Nodal	[120]
Ovarian cancer	HIF-2α knockdown	Substantially decreased the resistance of ovarian cancer stem cells to adriamycin; HIF-2α overexpression restored chemoresistance	[121]
Head and neck cancer	Periostin from CAFs	CAF-secreted periostin which activates protein kinase seven and enhances erlotinib chemoresistance	[122]
Glioma	Fibronectin	Culture with increasing fibronectin concentrations increased chemoresistance to carmustine in glioma stem cells	[123]

## 6. Autophagy

The stress of chemotherapy and hypoxia drives CSCs to alter metabolism and utilize alternative sources for energy production to maintain viability. One of the evolutionarily conserved mechanisms potentially implicated in enhanced survival and chemoresistance is autophagy. It is an essential housekeeper system that is important for cellular homeostasis. Intracellular proteins and organelles are degraded in autolysosomes that form upon the fusion of autophagosomes with lysosomes. Contents are degraded by lysosomal enzymes of the cathepsins family [124].

Autophagy was reported to enhance tumorigenesis and maintenance of stemness in multiple cancers, including colorectal, hepatocellular, CNS tumors, and melanoma [125,126,127,128]. Targeted blocking of autophagy in ex vivo studies has been shown to decrease stem cell properties of breast CSCs as well as a result in inhibited growth in vivo [129,130]. Chemotherapeutic agents such as oxaliplatin were shown to enrich the resistant CSCs pool in colorectal cancer [131]. Inhibiting autophagy in resistant CSCs dramatically increased susceptibility to conventional chemotherapeutic drugs and decreased stem cell properties. In glioblastoma, multiform targeted inhibition of the main autophagy regulator, ATG4B, has sensitized CSCs to radiotherapy. Smith et al., suggested that a particular subtype of autophagy, namely mitophagy, is required for the self-renewal of CSCs [132]. Mitophagy is the selective degradation of mitochondria to maintain low levels of oxidative phosphorylation hence leaving the cell dependent on glycolysis as a significant source of ATP. This is proposed to contribute to a slow self-renewing state. Anti-cancer medications induce autophagy, which in many cases promotes tumorigenesis and resistance to therapy. It was reported that autophagy promotes chemoresistance in ovarian CSCs treated with cisplatin [133].

Moreover, selective inhibition of autophagy in cisplatin-resistant CSCs resulted in enhanced apoptosis [134,135]. In chemoresistant non-small cell carcinoma xenograft tumors, combination treatment with autophagy inhibitor decreased the growth of the tumor and promoted apoptosis more effectively [136]. ER-positive breast cancer was also reported to be sensitized to tamoxifen after inhibition of autophagy [137]. Similar results were reported in previously enzalutamide-resistant prostate cancer and imatinib-resistant gastrointestinal stromal tumors [138,139]. The importance of autophagy microenvironment in chemoresistance of CSCs is demonstrated in Table 5.

**Table 5:** Summary of evidence for the role of autophagy microenvironment in chemoresistance of cancer stem cells.

Cancer Type	Intervention/Inhibitor	Experimental Evidence	References
CML	Lys05	Sensitized leukemia CSCs to tyrosine-kinase inhibitor treatment in vitro and in vivo	[140]
	Chloroquine RNAi	Combination with tyrosine kinase inhibitor treatment results in almost complete eradication of CML CSCs	[141]
	LV-320, ATG4B inhibitor	Sensitized imatinib-nonresponding progenitor cells to tyrosine-kinase inhibitors	[142]
Endometrial cancer	Chloroquine, 3-MA	Enhanced sensitivity of endometrial CSCs to paclitaxel	[143]
Colon cancer	36-077, a novel autophagy inhibitor	Combined treatment with 5-FU enhanced cytotoxic effect in cells expressing stemness markers	[144]
	Cdx1 siRNA	Sensitized colon CD44+ CSCs to the therapeutic effect of paclitaxel	[145]
	Knockdown of Atg5	Autophagy deficiency reversed the protective effect of autophagy and enhanced the action of oxaliplatin	[131]
	BNIP3L silencing (mitophagy)	Enhanced the sensitivity of CSCs to doxorubicin	[146]
	Atg5 silencing	Promoted apoptosis in previously resistant CSCs	[147]
Breast cancer	Chloroquine	Diminished DNA repair response in CSC, thereby increasing carboplatin sensitivity	[148]
		Reduced ‘stemness’ markers expression and sensitized ALDH+ CSCs to doxorubicin and docetaxel	[149]
Glioblastoma	Chloroquine	Significantly increased the therapeutic effect of bevacizumab	[150]
Ovarian cancer	Chloroquine CRISPR-Cas9	Enhanced cytotoxicity effect of carboplatin in vitro and decreased its tumorigenic abilities in vivo	[151]
Non-small cell lung carcinoma	Chloroquine	Combination treatment with cisplatin decreased the expression of stemness markers in CD133+ cells and enhanced its antitumor action	[136]
Gastric cancer	Chloroquine	Enhanced inhibitory action of 5-FU on CSCs	[152]
Bladder cancer	Chloroquine	Significantly increased apoptotic cell death in previously gemcitabine and mitomycin-resistant cells	[153]
Pancreatic cancer	shATG5, shATG7	Enhanced the sensitivity of pancreatic CSCs to gemcitabine	[154]

## 7. Epigenetics

Over the last decades, significant progress has been made in cancer genetics, identifying mutations and chromosomal alterations responsible for tumorigenesis and cancer progression [155]. A great body of evidence supports the idea that epigenetic changes such as DNA methylation, histone modifications, miRNAs, and chromatin remodeling might also be significant in the tumor initiation process [156,157,158]. Past advancements shifted the narrative to consider carcinogenesis a dynamic process of complex interactions between epigenetic and genetic factors. Since epigenetic alterations are important for the programming of cells during development, abnormal regulation of these processes was proposed to be involved in transforming normal stem cells to cancer stem cells with distinct properties such as enhanced chemotherapy resistance [159,160,161]. Chemotherapy-resistant cancer stem cells were commonly reported to have altered DNA methylation patterns. Therefore, hypomethylating agents were tested to restore sensitivity. Indeed, platinum-resistant ovarian cancer stem cells had significantly higher DNMT 3A and 3B expression than their non-resistant counterparts [162]. These cells were further re-sensitized to chemotherapy and essentially depleted in the presence of second-generation DNA methyltransferase inhibitor, SGI-110, supporting the role of epigenetic modification in the chemoresistance of CSCs.

miRNAs, small noncoding RNA molecules, are important regulators of gene expression and were also reported to be aberrantly expressed in cancer stem cells. For example, pancreatic cancer stem cells resistant to gemcitabine significantly downregulated specific clusters of miRNA 17-92 [163]. Overexpression of miR17-92 restored chemosensitivity to gemcitabine, abraxane, and 5-FU. Sensitivity to gemcitabine were also demonstrated in TSA, SAHA and for the pancreatic cancer specific inhibitors [164,165]. Interestingly, when differentiated cancer cells had been knocked down of miR 17-92 by an antisense inhibitor, they started to express stemness-related markers such as CD133 and ABC transporters and exhibited enhanced tumorigenesis. Another miRNA, miR-34a, was also found to be downregulated but in another cancer type. Overexpression of miR-34a in chemoresistant breast cancer stem cells showed to sensitize them to paclitaxel [166]. Authors suggest that this effect is due to the downregulation of Notch1 signaling pathway-related proteins, which indicates that epigenetic mechanisms may indirectly contribute to the chemoresistance of CSCs through the regulation of developmental pathways. These findings were consistent with another study where breast CSCs were re-sensitized to doxorubicin by ectopic expression of miR-34a [167]. Several other epigenetic mechanisms reported to be involved in the pathogenesis of chemoresistance of CSCs are summarized in Table 6.

**Table 6:** Summary of evidence for the role of epigenetics microenvironment in chemoresistance of cancer stem cells.

Cancer Type	Inhibitor	Experimental Evidence	References
Ovarian cancer	SGI-110	Low-dose DNA methyltransferase inhibitor limited the tumor-initiating capacity of stem cells and sensitized them to platinum-based therapy, promoting differentiation	[162]
Pancreatic cancer	Overexpression of miR-17-92	Increased sensitivity to gemcitabine, abraxane, and 5-FU in vitro, possibly through targeting Alk4	[163]
	TSA and SAHA	Enhanced action of gemcitabine against cancer stem cells	[164]
	UNC0638, G9a specific inhibitor	Sensitized previously resistant stem cells to gemcitabine	[165]
Breast cancer	miR-34a overexpression	Significantly increased cytotoxic effect of paclitaxel (PTX) on breast cancer stem cells, likely through downregulation of the Notch1 pathway	[166]
	Ectopic expression of miR-34a	Sensitized CSCs to doxorubicin	[167]
	microRNA-200 knockdown	Reversed paclitaxel resistance and stem cell properties	[168]
	miR-873	Adriamycin resistance was attenuated by activation of miR-873 signaling	[169]
	miR-128	Ectopic expression of miR-128 enhanced cytotoxicity of doxorubicin through the downregulation of Bmi-1 and ABCC5 protein levels	[170]
	Lnc-LBCS overexpression	Lnc-LBCS suppresses the chemoresistance of BCSCs in vitro and in vivo to gemcitabine and cisplatin	[171]
Adenoid cystic carcinoma	Vorinostat	Reduced the load of CSCs in vivo and in vitro alone and combination with cisplatin effectively depleted CSC	[172]
Glioblastoma multiforme	KDM2B knockdown	Enhanced cytotoxic effect in combination with both lomustine and etoposide augmented chemotherapy-induced apoptosis	[173]
Testicular cancer	GSKJ4	Treatment with the specific demethylase inhibitor resulted in the sensitization of the cells to cisplatin	[174]
CML	SIRT1 knockdown	Enhanced activity of imatinib, increased apoptosis in leukemia stem cells in vitro and in vivo	[175]

## 8. The Heterogeneity of CSCs, Hybrid Epithelial/Mesenchymal States and Stemness

Cancer stem cell heterogeneity can be found both in different cancer types and within the same tumor, causing the failure of drug treatment and disease relapse [176]. A better understanding of the CSC heterogeneity can promote the development of advanced therapeutic strategies against chemotherapy resistance [177]. Breast cancer tumor studies are a good example of the illustration of CSC plasticity and heterogeneity. It was shown that in the breast cancer tumor CD24^−^CD44^+^ and ALDH markers determine two distinct intra-tumor cell populations, where CD24^−^CD44^+^ is invasive with a mesenchymal-like state and ALDH is a more proliferative and epithelial-like state [178]. Another research group has found that ALDH can be identified in normal and breast cancer stem cells [179]. This CD44^+^CD24^−^/^low^ breast cancer cells phenotype was highly resistant to lapatinib [180]. Intriguingly, isoforms of CD44 can be found among a variety of breast cancer cell populations and correlated with different levels of cancer cell tumorigenicity [181]. Similarly, in prostate cancer, ALDH isoforms were detected in both primary tumors and metastasized prostate cancer cells [182]. Another example demonstrated that TGF-β signaling contributes to cancer stem cell heterogeneity in tumor cells. TGF- β responding cells of squamous carcinoma cells illustrated cisplatin resistance. In contrast, non-responding cell population induced proliferative and growth characteristics [183]. Later, Futakuchi et al., showed that TGF- β signaling pathway plays an important role in the characterization of CSC, proliferating cells, and therapy resistance [184]. The heterogeneity of the CSC is a complex phenomenon that needs solid markers and criteria to differentiate and identify the cancer stem cells in the tumor. EMT is a reversible evolutional process during which epithelial cells undertake temporary changes losing their phenotype and gaining a mesenchymal-like phenotype. The process when mesenchymal cells undergo opposite changes is called mesenchymal–epithelial transition (MET) [185]. The first concept of EMT was associated with embryogenesis [186]. However, recently the importance of EMT in various physiological and pathological processes, including wound healing, fibrosis, and cancer is highlighted [187]. Moreover, EMT has been proven to be involved in both resistance to therapy and the generation of CSC. Notably, the hybrid E/M state, when cancer cells are found to co-express both epithelial and mesenchymal markers, is linked with high metastatic potential and stemness [188]. For example, a pre-existing population of colorectal cancer cells with EMT-related ZEB2+ demonstrated stemness and mesenchymal characteristics driving resistance to chemotherapy [189]. Another study by Bontemps et al., published in 2022, showed that loss of CD24 by breast cancer cells facilitates stemness characteristics linked with hybrid E/M states, thereby fostering resistance to radio and chemotherapy [190]. Therefore, it might be concluded that hybrid E/M state CSC play a crucial role in therapy resistance; however, additional research is necessary.

## 9. Recent Developments in Combating CSCs Resistance

Over 19,000 online articles were published in PubMed in 2017–2019 only on CSCs and its resistance mechanisms to the various therapies demonstrating its advancements and significance to the research field. The strong self-renewal capacity of CSCs takes an important role in tumor relapse [191]. The existence of one CSC at the tumor site is capable to induce cancer development, relapse and metastasis. The latter changes lead to poor prognosis of the disease and play crucial role in resistance to therapies [191]. The mechanism of resistance of CSCs to traditional chemotherapy methods, through different ways, complicates destruction of cancer cells and tumors completely. Consequently, additional comprehensive studies should be done to understand novel mechanisms involved in CSCs resistance to therapies [192]. Russo et al., in 2019 suggested that de novo mutations might be caused by temporary enhancement of genetic instability during targeted therapy influenced by resistance. Their observation demonstrated temporal enhancement of mutagenesis, failure in DNA damage repair, in drug-tolerant tumor cells that endure and propagate with the EGFR/BRAF inhibition in colorectal cancer [193]. Das et al., reported in their study that CSCs number with CD133+ phenotype increases due to treatment by oxaliplatin. Cells carrying ESA+/CD44+/CD166+ stem cell surface markers demonstrated resistance to irinotecan. Both cases were reported in patients with colon cancer. Moreover, CSCs reported to show significant resistance to radiotherapy in HT-29 cells (human colon adenocarcinoma cells) [194]. Another study by Di Fiore et al., reported that significant increase in expression of ABC proteins, that function as a major CSCs mechanism of protection, play an important role in resistance to chemotherapy. This leads to the excretion of Hoechst dye and cytotoxic drugs that result in increase of resistance level to chemotherapy and cancer progression and poor prognosis [195].

Fumitremorgin C and verapamil drugs that are supposed to block ABC transporters demonstrate CSCs sensitivity in studies done using ovarian cancer [196]. Additionally, miRNAs play an important role in gene expression and are closely related to the biology of CSCs, as they regulate signaling pathways of stemness, EMT, cell differentiation processes, and tumorigenesis [195]. Di Fiore et al., claim that specific cluster of miRNAs found in tumor-initiating glioma cells (miR-302-367) might be used in future perspectives as a therapeutic agent since it demonstrated decreased level of growth of cervical cancer stem cells (CCSCs) through its involvement in regulation of cyclin D1 and AKT1 pathway [195]. As ECM components produce beneficial environment for CSCs, it is also possible to destroy their favorable niche through targeting therapy. Wang et al., reported that targeting hyaluronic acid, which is involved in enlargement of intra-tumoral pressure, might be beneficial against chemotherapy resistance and poor prognosis in patients [197], whereas Nawara et al., just recently (in 2021) argued that paclitaxel (PTX) if used in combination with other therapies might be beneficial against the resistance of cancer that are resistant to PTX alone. One of such examples is a study in which a combination of dasatinib with PTX resulted in partial reduction of breast CSCs proportion in the tumor, due to suppression of its self-renewal ability and hence, tumor growth [198]. Additionally, this combination demonstrated its inhibitory effect on pancreatic cancer cells by the phosphorylation of SRC, STAT3, AKT, and/or ERK protein [198]. Regardless of the enormous number of studies related to CSCs targeted therapy and variation of drug combination, it is important to enhance our in-depth understanding of these mechanisms in the future, in order to improve current therapies.

## 10. Future Perspectives

This article discusses the mechanism and potential re-sensitization of CSCs to the conventional chemotherapeutic drugs. CSCs can escape the cytotoxic effects of chemotherapeutic agents through various mechanisms, including those that were not included in this review. Mechanisms and examples discussed in this review may be useful in the identification of precise targeting strategies for various cancer types. A significant body of evidence and experimental data suggest that various developmental pathways and homeostasis mechanisms play a key role in chemoresistance development and mostly involve malfunction in pathway components. Therefore, novel, therapeutic approaches that aim to target particular pathway components for re-sensitization are an alternative approach that may have an important transitional effect, before new, advanced treatment strategies can be implemented. Studies provided in this review support the idea that CSCs can and should be re-sensitized using above mentioned pathway inhibitors, as an additional approach to improve outcomes in chemotherapy-resistant cancers. Other mechanisms, such as enhanced DNA repair response and CSCs metabolism, may be further explored to develop specific therapy and improve the survival of patients with highly aggressive chemoresistant cancers.

## Data Availability

Not applicable.
